# Spectroscopic Characterization and Biological Activity of Mixed Ligand Complexes of Ni(II) with 1,10-Phenanthroline and Heterocyclic Schiff Bases

**DOI:** 10.1155/2012/948534

**Published:** 2012-10-02

**Authors:** Y. Prashanthi, K. Kiranmai, Ira, Sathish kumar K, Vijay kumar Chityala, Shivaraj

**Affiliations:** ^1^Department of Chemistry, Osmania University, Hyderabad 500 007, India; ^2^Indian Institute of Chemical Technology, Hyderabad 500 007, India; ^3^Centre for Cellular and Molecular Biology, Hyderabad 500 007, India

## Abstract

Mixed ligand complexes of Ni(II) with 1,10-phenanthroline (1,10-Phen) and Schiff bases L_1_(MIIMP); L_2_(CMIIMP); L_3_(EMIIMP); L_4_(MIIMNP); L_5_(MEMIIMP); L_6_(BMIIMP); L_7_(MMIIMP); L_8_(MIIBD) have been synthesized. These metal chelates have been characterized by elemental analysis, IR, ^1^H-NMR, ^13^C-NMR, Mass, UV-Vis, magnetic moments, and thermogravimetric (TG&DTA) analysis. Spectral data showed that the 1,10-phenanthroline act as neutral bidentate ligand coordinating to the metal ion through two nitrogen donor atoms and Schiff bases acts as monobasic bidentate coordinating through NO donor atoms. All Ni(II) complexes appear to have an octahedral geometry. The antimicrobial activity of mixed ligand complexes has been studied by screening against various microorganisms, it is observed that the activity enhances upon coordination. The DNA binding studies have been investigated by UV-Vis spectroscopy, and the experimental results indicate that these complexes bind to CT DNA with the intrinsic binding constant K_*b*_ = 2.5 ± 0.2 × 10^5^ M^−1^. MTT is used to test the anticancer effect of the complexes with HL60 tumor cell. The inhibition ratio was accelerated by increasing the dosage, and it had significant positive correlation with the medication dosage.

## 1. Introduction 

One of the major applications of the transition metal complexes is their medical testing as antibacterial and antitumor agents aiming toward the discovery of an effective and safe therapeutic regimen for the treatment of bacterial infections and cancers. In addition, many Schiff base complexes with metals have also provoked wide interest because they possess a diverse spectrum of biological and pharmaceutical activities, including antitumor, antioxidative, antifungal, and antibacterial activities [1–10]. Schiff bases and their complexes have been used as biological models to understand the structures of biomolecules and biological processes [11, 12]. The study of ternary complexes involving an aromatic Schiff base and 1,10-phenanthroline has been studied extensively [13]. To design effective chemotherapeutic agents and better anticancer drugs, it is essential to explore the interactions of metal complexes with DNA [14]. Moreover, it is well known that some drug activities, when administered as metal complexes, are being increased, and several Schiff base complexes have also been shown to inhibit tumor growth. The incorporation of transition metal into Schiff bases enhances the biological activity of the ligand and decreases the cytotoxic effects of both the metal ion and ligand on the host [15]. 

In view of the wide biological activities exhibited by isoxazoles and their derivatives which include antibacterial, anticancer, anti-HIV and have applications as pesticides and insecticides, it is meaningful to extend these studies on metal complexes containing isoxazole Schiff bases. Nickel is recognized as an essential trace element for bacteria, plants, animals, and humans though the role of this metal in animal biochemistry is still not well defined.

Previously, we have reported our interesting results on the synthesis and antimicrobial activities of binary complexes of isoxazole Schiff bases [16]. Encouraged by this result, we turned our attention towards the synthesis, biological studies, and DNA binding studies of ternary complexes. A thorough literature survey reveals that not much work has been done on the synthesis and biological studies of ternary complexes of isoxazole Schiff bases. 

Hence, we are tempted to synthesize biologically important ternary Schiff base complexes. Herein, it is reported that the synthesis, spectral characterization and antimicrobial studies of ternary Schiff base complexes, and their interaction with DNA. 

The following ligands which are selected in the present investigation are

1. 2-{[(5′-Methyl-3′-isoxazolyl)imino]methyl}phenol (MIIMP) (L_1_),
2. 4-Chloro-2-{[(5′-Methyl-3′-isoxazolyl)imino]methyl}phenol(CMIIMP) (L_2_),
3. 2-Ethoxy-6-{[(5′-Methyl-3′-isoxazolyl)imino]methyl}phenol(EMIIMP) (L_3_),
4. 2-{[(5′-Methyl-3′-isoxazolyl)imino] methyl}-4-nitrophenol(MIIMNP) (L_4_),
5. 4-Methyl-2-{[(5′-Methyl-3′-isoxazolyl)imino] methyl}phenol(MEMIIMP) (L_5_),
6. 4-Bromo-2-{[(5′-Methyl-3′-isoxazolyl)imino] methyl}phenol(BMIIMP) (L_6_),
7. 5-Methoxy-2-{[(5′-Methyl-3′-isoxazolyl)imino] methyl}phenol(MMIIMP) (L_7_),
8. 4-{[(5′-Methyl-3′-isoxazolyl)imino]}-1,3-benzenediol (MIIBD) (L_8_).

## 2. Experimental 

### 2.1. Materials and Methods

^1^H NMR spectra of the ligands were recorded at 200 MHz and 300 MHz on Varian Gemini Unity Spectrometer using TMS as an internal standard.^ 13^C NMR spectra were recorded at 100.6 MHz on Varian Gemini Spectrometer. The EI mass spectra were recorded on a VG micro mass 7070-H Instrument, ESI MS spectra were recorded on VG AUTOSPEC mass spectrometer. IR spectra of the ligands and complexes were recorded using KBr pellets in the range (4000–400 cm^−1^) on Perkin-Elmer Infrared model 337. Electronic spectra of metal complexes in DMSO were recorded on Schimadzu UV-VIS 1601 spectrophotometer. Magnetic susceptibilities of the complexes were determined on Gouy balance model 7550 using Hg [Co(NCS)_4_] as standard. The diamagnetic corrections of the complexes were computed using Pascal's constants. TGA of complexes were carried on Mettler Toledo Star system in the temperature range of 0–1000°C. Melting points of the ligands and decomposition temperature of complexes were determined on Polmon instrument (model no. MP-96). The conductivity measurements were measured in DMSO solutions (0.001 M) using Digisun Electronic Digital conductivity meter of model: DI-909 having a dip-type cell calibrated with KCl solution. The percentage composition of C, H, N for the complexes and necessary ligands were determined by using microanalytical techniques on Perkin Elmer 240C (USA) elemental analyzer. The EPR spectra of the Copper complexes were recorded on EPR Varian-E-112 at RT. The percent composition of metal ions in solid metal complexes was determined by atomic absorption spectrophotometer.

### 2.2. Synthesis of Schiff Bases (L_1_–L_8_) (General Method)

The Schiff bases, namely L_1_, L_2_, L_3_, L_4_, L_5_, L_6_, L_7_, and L_8_, were prepared by the condensation of 3-amino-5-methyl isoxazole (5 mmol) with the respective salicylaldehydes (5 mmol) was taken in methanol and refluxed for 2 h. The colored Schiff bases obtained were recrystallized from methanol and petroleum ether (8 : 2). Purity of the compound was checked by TLC. Yield: 80–85%. 

### 2.3. Synthesis of [Ni(L_1_/L_2_/L_3_/L_4_/L_5_/L_6_/L_7_/L_8_)(1,10-phen)(H_2_O)_2_]Cl Complexes

The Nickel(II) complexes were prepared by mixing the appropriate molar quantity of ligands and nickel salt using the following procedure. An ethanolic solution of Schiff base L_1_/L_2_/L_3_/L_4_/L_5_/L_6_/L_7_/L_8_ (10 mmol) was reflux with the ethanolic solution of Nickel(II) chloride (10 mmol) for ca 3 h. To the above mixture, an ethanolic solution of 1,10-phenanthroline(10 mmol) was added, and the reflux was continued for ca 1 h. The colored solid product formed was filtered, washed with ethanol, and dried in vacuo. 

### 2.4. Antimicrobial Screening

The ligands and their metal complexes were screened against bacteria and fungi. Anti-bacterial screening was done by the paper disc method (Kirby-Bauer method) [17]. The bacterial organisms used are *Pseudomonas aeruginosa *(gram +ve) and *Escherichia coli* (gram −ve). Cultures of test organisms were maintained in nutrient agar media and subcultured in Petri dishes prior to testing. The fungal organisms used are *Aspergillus niger* and *Rhizoctonia solani*. Cultures were maintained on potato dextrose agar slants and subcultured in petri dishes prior to testing. 

### 2.5. DNA Binding Studies

 Absorption spectra were recorded on Jasco V-530 UV-visible spectrophotometer using 1 cm quartz-cuvettes. Absorption titrations were performed by keeping the concentration of the complex constant (10 *μ*M) and by varying [CT DNA] from 0–11 *μ*M. For [Ni (1,10-phen) (EMIIMP) (H_2_O)_2_]Cl complex, the binding constant (K_*b*_), has been determined from the spectroscopic titration data using the following equation [18]:

(1) [DNA](εa−εf)=  [DNA](εb−εf)+1Kb(εb−εf).

The “apparent” extinction coefficient (*ε*_*a*_) was obtained by calculating A_obsd_/[Ni]. The terms *ε*_*f*_ and *ε*_*b*_ correspond to the extinction coefficients of free (unbound) and the fully bound complex, respectively. From plot of [DNA]/(*ε*_*a*_ − *ε*_*f*_) versus [DNA] will give a slope 1/(*ε*_*b*_ − *ε*_*f*_) and an intercept 1/K_*b*_(*ε*_*b*_ − *ε*_*f*_). K_*b*_ is the ratio of the slope and the intercept.

### 2.6. Anticancer Activity

The Ni(II) complexes are screened against HL 60 cells. The activity among these complexes are in the range of IC_50_ (*μ*g/mL) are 35.48 ± 1.12. 

### 2.7. Methodology

#### 2.7.1. Cell Line

Human promyelocytic leukemia (HL60) cells were cultured in RPMI-1640. The media were supplemented with 10% heat-inactivated FCS, 1 mM NaHCO_3_, 200 mM L-glutamine, and penicillin-streptomycin in a humidified atmosphere of 95%, 5% CO_2_ at 37°C.

#### 2.7.2. Test Concentrations

Initially, the stock concentrations were prepared by dissolving 8 mg of each test compound in 1 mL of DMSO and further diluted to obtain a experimental stock solution of 200 *μ*g/mL (25 *μ*L of initial stock solution was diluted to 1 mL). Different aliquots of experimental stock were added to the cultured cells in the medium (final volume of 200 *μ*L) to attain the required test concentrations of 10, 20 40, 60, 80, and 100 *μ*g/mL. 

## 3. Results and Discussion

### 3.1. Characterization of Complexes

All the complexes are stable in air and have high melting points. They are freely soluble in DMSO and DMF and sparingly soluble in methanol and ethanol. The metal complexes were characterized by elemental analysis, molar conductivities, TG, DTA, IR, UV-Vis, ^1^H NMR,^ 13^C NMR, and Mass spectra. The analytical data of the complexes are in agreement with the experimental data. The values reveal that the metal to ligand ratio is 1 : 1 : 1 and are presented in Table 1.

### 3.2. Thermal Analysis

 In the present investigation, the following thermoanalytical methods have been used.

**(1) Thermogravimetric analysis (TGA) and (2) Differential Thermal Analysis (DTA)**

From the thermograms of these complexes, it is concluded that the coordinated water molecules are eliminated in the temperature range of 60–1600°C. And the ligands gradually decompose to their corresponding metal oxides at higher temperatures. Presence of water molecules is further confirmed by the endothermic bands observed in the respective DTA curve in the temperature region where the TG curves should lose weight. In addition to the endothermic bands, the DTA curves of complexes also show exothermic bands. These bands appeared at higher temperatures which represent phase transition, oxidation and/or decomposition of the compound.

**Figure 1:**
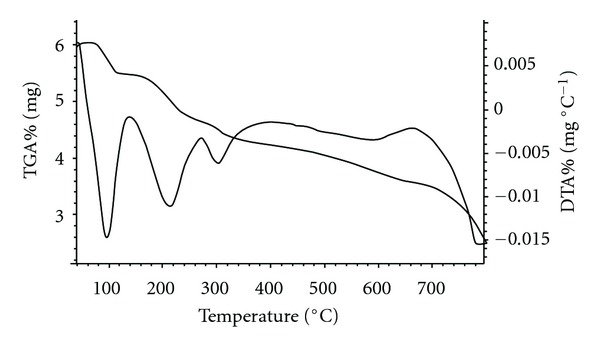
TGA Spectrum of [Ni(1,10-phen)(MIIMP)(H_2_O)_2_]Cl.

The thermal decomposition process of [Ni(1,10-phen)(MIIMP)(H_2_O)_2_]Cl complex (Figure 1) can be divided into three stages. The first stage occurs in the range of 80–1300°C having mass loss of 12.05% (calculated 12.88%) which corresponds to the loss of two moles of coordinated water and chloride ion. In continuation to the first stage, the degradation stages occur in the range of 170–3300°C having mass loss of 38.64% (calculated 39.12%) shows partial decomposition of the ligand. The degradation stage in the range of 330 to 600°C with an estimated mass loss of 31.98% (calculated 32.48%). This mass loss corresponds to the pyrolysis of ligand molecules leaving NiO as a residue.

### 3.3. IR Spectra

The IR spectral data for the complexes are summarized in Table 2. A broadband at 3319–3445  cm^−1^ due to phenolic OH group of free Schiff base disappear in their metal complexes indicating coordination through the phenolic oxygen & bands at 1601–1616 cm^−1^ in the free ligands are due to azomethine group, which is shifted to lower frequencies in the spectra of the complexes (1570–1600 cm^−1^) indicating involvement of azomethine nitrogen in coordination to the metal. The medium intensity bands observed for all ligands in the range 1350–1358 cm^−1^due to the phenolic stretching frequency, shifted to lower frequency by 10–20 cm^−1^ in the mixed-ligand complexes suggesting involvement of the oxygen atom of the ***ν***C–O moiety in coordination [19]. The presence of coordinated water molecules are observed by broadbands around 3342–3535 cm^−1^ which can further confirmed by bands observed at 765–780 cm^−1^ [20].

Shifting of bands in the region 1500–1300 cm^−1^ of the free phenanthroline ligand is observed in the spectra. In particular, the peaks corresponding to the ring stretching frequencies ***ν***C=C and ***ν***C=N at 1505 and 1421 cm^−1^ undergo shifting to higher frequencies at 1520 and 1427 cm^−1^, indicating the coordination of the “1,10-phen” nitrogen atoms to the metal ion [21]. In the region 800–600 cm^−1^, characteristic out-of-plane hydrogen bending modes of free phenanthroline at 855 and 738 cm^−1^, shift to frequencies of 850 and 728 cm^−1^, respectively. Similarly, the far-infrared region of the spectra shows new peaks at 510–417 cm^−1^ due to the ***ν***M–O and ***ν***M–N vibrations, respectively [22].

### 3.4. Electronic Absorption Spectra and Magnetic Measurements

**Scheme 1:**
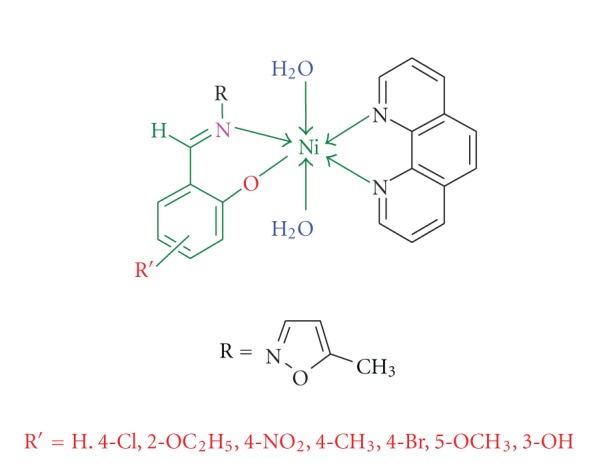
Proposed structure of the Nickel complexes.

Absorption band assignments and magnetic measurement data of the Ni(II) complexes are given in Table 3. The electronic spectra of present Ni(II) complexes show three bands at 646–831, 506–641, and 356–414 nm, presumably due to the three spin-allowed transitions. ^3^A_2g_ (F) → ^3^T_2g_, ^3^A_2g_ (F) → ^3^T_1g_ (F) and ^3^A_2g_ (F) → ^3^T_1g_ (P) transitions for octahedral structure (Scheme 1). 

The Ni(II) complexes are found to have magnetic moments in the range 2.81–3.2 B.M., which are well within the range expected for octahedral Ni(II) complexes. The higher magnetic moments when compared to the spin-only value 2.8 B.M., may be attributed to the slight mixing of a multiplet excited state in which spin-orbit coupling is appreciable [23].

### 3.5. FAB Mass Spectra

**Figure 2:**
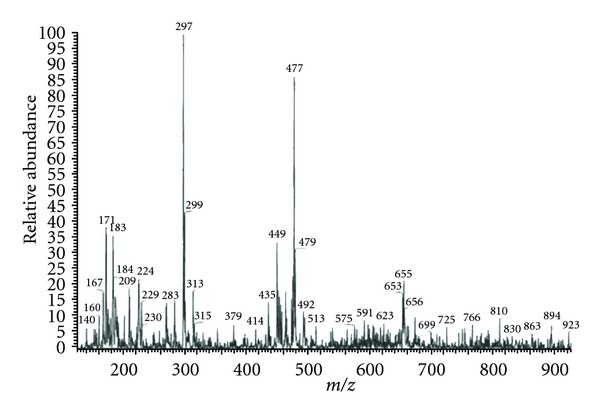
Mass Spectrum of [Ni(1,10-phen)(MIIMP)(H_2_O)_2_]Cl.

The FAB spectra of [Ni(1,10-phen)(MIIMP)(H_2_O)_2_]Cl have been depicted and reproduced in Figure 2. The spectrum showed a molecular ion peak M^+^ at m/z 476 that is equivalent to its molecular weight [Ni(1,10-phen)(MIIMP)(H_2_O)_2_]Cl.

The FAB spectra of [Ni(1,10-phen)(MIIMNP)(H_2_O)_2_]Cl showed a molecular ion M^+^ peak at m/z 543 that is equivalent to its molecular weight [(Ni(1,10-Phen)(MIIMNP)(H_2_O)_2_) + Na]^+^. The molecular ion by the loss of two water molecules gave a fragment ion peak at m/z 379. All these fragments leading to the formation of the species [ML]^+^ which undergoes demetallation to form the species [L + H]^+^at m/z 248. All these spectral data which confirm the ratio of complexes are in 1 : 1 : 1.

## 4. Molecular Modeling Studies

**Figure 3:**
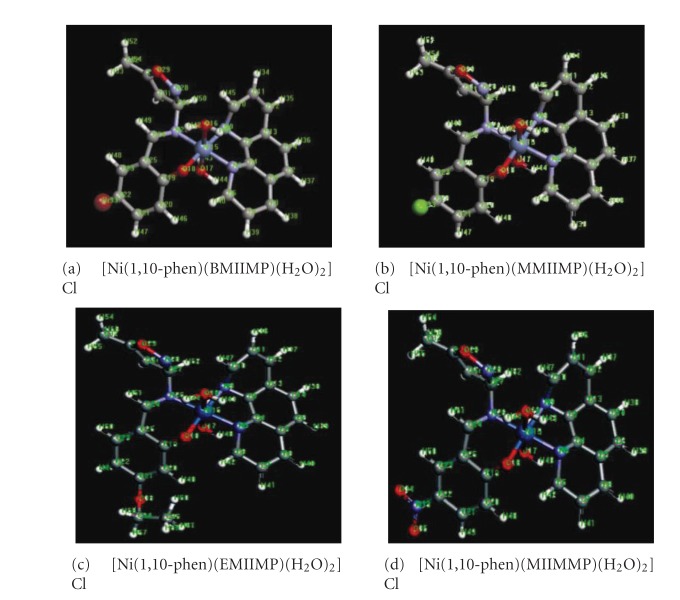
The optimized structural geometry of Ni(II) complexes.

In the absence of X-ray crystal structure data the 3-dimensional structure of the molecules can not be entirely unambiguous. The configuration possible for the Ni(II) complexes were evaluated using the semiempirical and the density functional theory calculations, respectively. The most stable structure with minimum energy among the possible ones is judged as the most probable structure as shown in Figure 3.

## 5. Antimicrobial Activity

The ligands and their metal complexes were screened against bacteria and fungi. Antibacterial screening was done by the paper disc method (Kirby-Bauer method). Ternary Ni(II) complexes with 1,10-phenanthroline and MIIMP, CMIIMP, EMIIMP, MIIMNP, MEMIIMP, MMIIMP, BMIIMP, and MIIBD were screened against bacteria and fungi, and the results obtained are presented in Table 4. It is observed that the activity of ternary complexes is more compared to their corresponding binary complexes. This result is expected since the complexes possess a greater planar area and *π*-systems which make stacking more strongly. The variation in the activity of different complexes against different organisms depend either on the impermeability of the cells of the microbes or difference in ribosomes of microbial cells [24]. Comparison of the biological activity of the synthesized compounds with some known antibiotics (gentamycin) shows generally the free Schiff base ligand, and some of its complexes exhibit better activity than these antibiotics or comparable effect.

## 6. DNA Binding Studies

**Figure 4:**
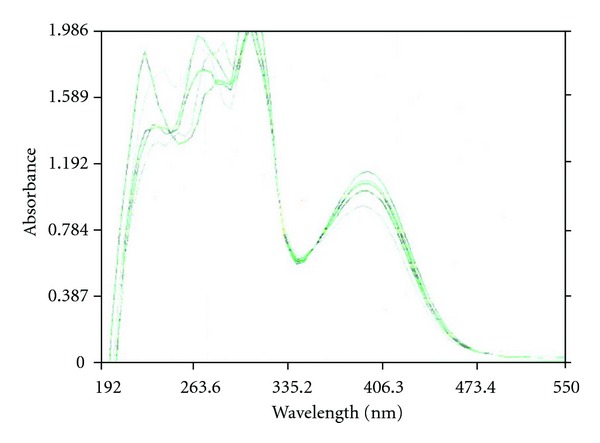
UV-Vis absorption Spectra of [Ni(1,10-phen)(EMIIMP)(H_2_O)_2_]Cl (10 *μ*M) in the presence of increasing amounts of CT-DNA; [DNA] = 0, 10, 20, 30, 40, 50 *μ*M.

The binding of the [Ni(1,10-phen)(EMIIMP)(H_2_O)_2_]Cl to the CT DNA (Calf Thymus DNA) has been studied by electronic absorption spectroscopy in Figure 4. The electronic absorption spectra of [Ni(1,10-phen)(EMIIMP)(H_2_O)_2_]Cl in the absence and presence of CT DNA have been recorded in Tris HCl buffer at 300 K. Increasing concentration of CT DNA results in hypochromism and a red shift in the UV-Vis spectrum of complex. These spectral characteristics suggest that the π* orbital of the intercalated ligand couples with the *π* orbital of base pairs, thus decreasing the *π*-π* transition energy and further resulting in the red shift [25]. Coupling of the *π* partially filled by electrons decreases the transition probabilities and concomitantly results in hypochromism. In order to compare the binding strength of the complexes with CT DNA, the intrinsic binding constants K_*b*_ are obtained by monitoring the changes in absorbance with increasing concentration of DNA. K_*b*_ is obtained from the ratio of the slope to the intercept from plots of [DNA]/(*ε*_*a*_ − *ε*_*f*_) versus [DNA]. The K_*b*_ value is in the order of 2.5 ± 0.2 × 10^5^ M^−1^. The high K_*b*_ value is to the presence of planar structure of phen ligand which facilitates the groove binding/stacking with base pairs [26].

## 7. Anticancer Activity

All the synthesized nickel complexes were screened for their cytotoxicity (HL60 cell). From the data, it was observed that the complexes displayed their cytotoxic activities as IC_50_ (*μ*g/mL) against human promyelocytic leukemia (HL60 cells), the IC_50 _values of all the nickel complexes are listed in Table 5.

**Cell Morphological Assessment**

**Figure 5:**
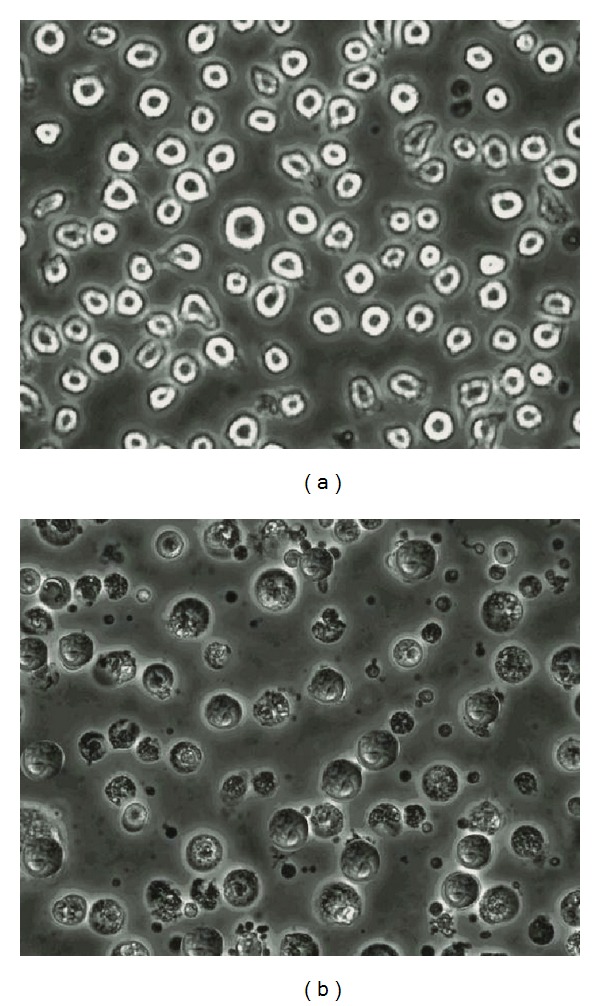
Morphological changes in HL60 cells (a) control cells with intact nuclei; (b) cell membrane blebbing, nuclear fragmentation, and chromatin condensation in presence of [Ni(1,10-phen)(EMIIMP)(H_2_O)_2_]Cl.

The morphological abnormalities were studied under a phase-contrast microscope. Cells treated for 24 h showed obvious morphological changes, with chromatin condensation, fragmentation, and formation of apoptotic bodies. However, the control group (without test compound) showed normal healthy shape with intact nuclei and without any abnormalities is shown in Figure 5. Most of the treated cells in all the test concentrations exhibited similar symptoms of apoptosis but the damage was severe in the cells exposed to highest concentration.

## 8. Conclusions 

New mixed ligands and their ternary Ni(II) complexes have been designed, synthesized, and characterized. Based on analytical, conductivity, magnetic data, infrared, and electronic spectral data, they adopt octahedral geometry around Nickel(II). The TG investigations were able to evaluate the presence of water molecules in the coordination sphere. Based on these observations, metal ion coordinates through phenolate oxygen, azomethine nitrogen of Schiff bases, and nitrogen atoms of phenanthroline. The antimicrobial study reveals that some of nickel complexes show better activity than the known antibiotic. The antitumor activity of complexes displayed good cytotoxic activities against human promyelocytic leukemia (HL60 cells). DNA binding studies of complexes reveal that presence of high K_*b*_ value facilitates the groove binding/stacking with base pairs. 

## Figures and Tables

**Table 1:** Analytical data for Ternary Ni(II) complexes.

Compound	Formula	M.Wt	C %	H %	N %	M %
[Ni (1,10-phen) (MIIMP) (H_2_O)_2_]Cl	[NiC_23_H_21_N_4_O_4_Cl]	511.2	53.99 (54.37)	4.10 (4.01)	10.95 (10.02)	11.48 (11.06)
[Ni (1,10-phen) (CMIIMP) (H_2_O)_2_]Cl	[NiCH_20_N_4_O_4_Cl_2_]	545.7	50.57 (50.00)	3.66 (3.27)	10.26 (10.17)	11.75 (11.27)
[Ni (1,10-phen) (EMIIMP) (H_2_O)_2_]Cl	[NiC_25_H_25_N_4_O_5_Cl]	555.2	54.03 (53.38)	4.50 (4.41)	10.08 (10.00)	10.57 (10.27)
[Ni (1,10-phen) (MIIMNP) (H_2_O)_2_]Cl	[NiC_23_H_20_N_5_O_6_Cl]	556.2	49.62 (51.27)	3.59 (3.24)	12.58 (12.74)	10.55 (10.72)
[Ni (1,10-phen) (MEMIIMP) (H_2_O)_2_]Cl	[NiC_24_H_23_N_4_O_4_Cl]	525.2	54.83 (54.37)	4.37 (4.21)	10.66 (10.02)	11.17 (10.95)
[Ni (1,10-phen) (MMIIMP) (H_2_O)_2_]Cl	[NiC_24_H_23_N_4_O_5_Cl]	541.2	53.21 (52.59)	4.24 (4.20)	10.34 (10.97)	10.84 (10.37)
[Ni (1,10-phen) (BMIIMP) (H_2_O)_2_]Cl	[NiC_23_H_20_N_4_O_4_BrCl]	590.2	46.76 (46.20)	3.38 (3.30)	9.48 (9.00)	9.93 (9.35)
[Ni (1,10-phen) (MIIBD) (H_2_O)_2_]Cl	[NiC_23_H_21_N_4_O_5_Cl]	527.2	52.35 (52.00)	3.98 (3.20)	10.62 (10.20)	11.13 (11.02)

^∗^The values mentioned within the bracket are calculated.

**Table 2:** IR Absorption frequencies of Ternary Ni(II) Complexes.

Complex	*ν* OH	*ν* C=N	*ν* C–O	Coordinated water	*ν* M–O	*ν* M–N
[Ni (1,10-phen) (MIIMP) (H_2_O)_2_]Cl	—	1616	1342	765	525	473
[Ni (1,10-phen) (CMIIMP) (H_2_O)_2_]Cl	—	1570	1340	785	510	424
[Ni (1,10-phen) (EMIIMP) (H_2_O)_2_]Cl	—	1582	1342	780	540	429
[Ni (1,10-phen) (MIIMNP) (H_2_O)_2_]Cl	—	1604	1344	780	535	417
[Ni (1,10-phen) (MEMIIMP) (H_2_O)_2_]Cl	—	1624	1343	765	525	473
[Ni (1,10-phen) (MMIIMP) (H_2_O)_2_]Cl	—	1620	1341	785	510	424
[Ni (1,10-phen) (BMIIMP) (H_2_O)_2_]Cl	—	1587	1342	779	540	429
[Ni (1,10-phen) (MIIBD) (H_2_O)_2_]Cl	—	1616	1344	780	535	417

**Table 3:** Magnetic moments and electronic spectral data for ternary complexes.

Complex	Frequency in nm (*ϵ* = 10^3^ M^−1^ cm^−1^)			*μ*_ eff_ B.M
[Ni (1,10-phen) (MIIMP) (H_2_O)_2_]Cl	671 (0.092)	521 (0.058)	399 (0.067)	3.21
[Ni (1,10-phen) (CMIIMP) (H_2_O)_2_]Cl	746 (0.083)	552 (0.047)	414 (0.056)	2.81
[Ni (1,10-phen) (EMIIMP) (H_2_O)_2_]Cl	646 (0.096)	524 (0.065)	380 (0.039)	3.00
[Ni (1,10-phen) (MIIMNP) (H_2_O)_2_]Cl	831 (0.072)	641 (0.051)	392 (0.043)	3.20
[Ni (1,10-phen) (MEMIIMP) (H_2_O)_2_]Cl	686 (0.094)	542 (0.072)	396 (0.051)	3.12
[Ni (1,10-phen) (MMIIMP) (H_2_O)_2_]Cl	699 (0.085)	552 (0.063)	356 (0.049)	3.15
[Ni (1,10-phen) (BMIIMP) (H_2_O)_2_]Cl	688 (0.079)	506 (0.058)	370 (0.041)	3.05
[Ni (1,10-phen) (MIIBD) (H_2_O)_2_]Cl	692 (0.093)	588 (0.074)	408 (0.053)	3.20

**Table 4:** Antimicrobial activity of ternary complexes.

Complex	*E. coli*	*P. aeruginosa*	*R. oryzae*	*A. niger*
MIIMP	+	+	−	+
[Ni (MIIMP)_2_ (H_2_O)_2_]	+	+	+	+
[Ni (1,10-phen) (MIIMP) (H_2_O)_2_]Cl	++	++	+	++
CMIIMP	+	+	+	+
[Ni (CMIIMP)_2_ (H_2_O)_2_]	+	−	+	−
[Ni (1,10-phen) (CMIIMP) (H_2_O)_2_]Cl	++	+	+	+
EMIIMP	+	+	+	+
[Ni (EMIIMP)_2_ (H_2_O)_2_]	++	++	++	++
[Ni (1,10-phen) (EMIIMP) (H_2_O)_2_]Cl	+++	++	+	++
MIIMNP	+	+	+	+
[Ni (MIIMNP)_2_ (H_2_O)_2_]	++	++	++	++
[Ni (1,10-phen) (MIIMNP) (H_2_O)_2_]Cl	++	+	+	++
MEMIIMP	+	−	−	+
[Ni (MEMIIMP)_2_ (H_2_O)_2_]	+	+	+	+
[Ni (1,10-phen) (MEMIIMP) (H_2_O)_2_]Cl	+	+	+	+
MIIMMP	+	+	+	+
[Ni (MIIMMP)_2_ (H_2_O)_2_]	++	+	−	+
[Ni (1,10-phen) (MMIIMP) (H_2_O)_2_]Cl	+	++	+	+
BMIIMP	+	−	−	−
[Ni (BMIIMP)_2_ (H_2_O)_2_]	++	+	+	+
[Ni (1,10-phen) (BMIIMP) (H_2_O)_2_]Cl	+++	+	+	−
MIIBD	+	+	+	+
[Ni (MIIBD)_2_ (H_2_O)_2_]	++	+	+	−
[Ni (1,10-phen) (MIIBD) (H_2_O)_2_]Cl	++	+++	+	+
Gentamycin	++	++	−	−

Highly active: +++ (inhibition zone > 15 mm); moderatively active: ++ (inhibition zone > 10 mm); slightly active: + (inhibition zone > 5 mm); inactive: − (inhibition zone < 5 mm ).

**Table 5:** IC_50_ range of Ni(II) complexes for HeLa cells.

Complex	IC_50_ (*μ*g/mL)
[Ni (1,10-phen) (MIIMP) (H_2_O)_2_]Cl	65.48 ± 0.18
[Ni (1,10-phen) (CMIIMP) (H_2_O)_2_]Cl	68.32 ± 0.21
[Ni (1,10-phen) (EMIIMP) (H_2_O)_2_]Cl	35.48 ± 0.12
[Ni (1,10-phen) (MIIMNP) (H_2_O)_2_]Cl	50.84 ± 0.43
[Ni (1,10-phen) (MEMIIMP) (H_2_O)_2_]Cl	47.31 ± 0.85
[Ni (1,10-phen) (MMIIMP) (H_2_O)_2_]Cl	71.25 ± 0.42
[Ni (1,10-phen) (BMIIMP) (H_2_O)_2_]Cl	82.30 ± 0.24
[Ni (1,10-phen) (MIIBD) (H_2_O)_2_]Cl	52.51 ± 0.72
